# Genotyping of *CYP2C9* and *VKORC1* in the Arabic Population of Al-Ahsa, Saudi Arabia

**DOI:** 10.1155/2013/315980

**Published:** 2013-03-17

**Authors:** Abdullah M. Alzahrani, Georgia Ragia, Hamza Hanieh, Vangelis G. Manolopoulos

**Affiliations:** ^1^Biological Sciences Department, College of Science, King Faisal University, Hofouf 31982, Saudi Arabia; ^2^Laboratory of Pharmacology, Medical School, Democritus University of Thrace, Alexandroupolis 68100, Greece

## Abstract

Polymorphisms in the genes encoding CYP2C9 enzyme and VKORC1 reductase significantly influence the dose variability of coumarinic oral anticoagulants (COAs). Substantial inter- and intraethnic variability exists in the frequencies of *CYP2C9* ^**∗**^2 and ^**∗**^3 and *VKORC1* –1639A alleles. However, the prevalence of *CYP2C9* and *VKORC1* genetic variants is less characterized in Arab populations. A total of 131 healthy adult subjects from the Al-Ahsa region of Saudi Arabia were genotyped for the *CYP2C9* ^**∗**^2 and ^**∗**^3 and *VKORC1* –1639G>A polymorphisms by PCR-RFLP method. The frequencies of the *CYP2C9* ^**∗**^2 and ^**∗**^3 and *VKORC1* –1639A alleles were 13.3%, 2.3%, and 42.4%, respectively, with no subjects carrying 2 defective alleles. The frequencies of the *CYP2C9* ^**∗**^3 and *VKORC1* –1639A alleles were significantly lower than those reported in different Arabian populations. None of the subjects with the *VKORC1* –1639AA genotype were carriers of *CYP2C9* ^**∗**^1/^**∗**^3 genotypes that lead to sensitivity to COAs therapy. The low frequency of the *CYP2C9* ^**∗**^3 allele combined with the absence of subjects carrying 2 defective *CYP2C9* alleles suggests that, in this specific population, pharmacogenetic COAs dosing may mostly rely upon *VKORC1* genotyping.

## 1. Introduction

Pharmacogenomics is the first step towards personalized medicine and is a promising field of investigation that may explain some of the interindividual variations in responses to various classes of drugs [1, 2]. Pharmacogenomics can be applied to all fields of medicine. In particular, recent advances in genotype-phenotype associations in cardiology point towards the application of pharmacogenomics to oral coumarinic anticoagulants (COAs), including warfarin, acenocoumarol, and phenprocoumon, in routine clinical practice [3].

 Among other factors, interindividual COAs dose variability is significantly influenced by variations in the genes encoding two enzymes: cytochrome P450 2C9 (CYP2C9), the enzyme that metabolizes COAs, and vitamin K epoxide reductase (VKORC1), the pharmacologic target of these drugs [3]. 

Polymorphisms in the *CYP2C9* gene seriously affect the enzymatic activity of the encoded CYP2C9 protein. Based on phenotype, populations can be divided into extensive (EM), intermediate (IM), and poor metabolizers (PM), and more than 35 different allelic variants have been identified in the *CYP2C9* gene [4]. Among these alleles, the CYP2C9 *2 (rs1799853) and *3 (rs1057910) variants, which reduce CYP2C9 enzymatic activity, allow for the prediction of more than 85% of PMs [5]. In addition, it has recently been shown that *VKORC1* gene polymorphisms also affect COAs dosing requirements [6]. The *VKORC1* –1639G>A polymorphism (rs9923231) is located in the promoter of the *VKORC1* gene and results in reduced promoter activity and lower mRNA levels, which lead to lower levels of synthesized protein and eventually the reduced production of active clotting factors in subjects with the AA genotype [6]. 

Whereas the CYP2C9 *2 and *3 alleles affect coumarin pharmacokinetics, the *VKORC1* –1639G>A polymorphism affects the pharmacodynamic response to coumarins [3]. It has been reported that polymorphisms in the *CYP2C9* and *VKORC1* genes together account for 35%–50% of the variability in COAs dose requirements for initiation and maintenance [7]. Moreover, carriers of the CYP2C9 *2 or *3 alleles and the *VKORC1* –1639G>A polymorphism are at higher risk for bleeding and require lower mean daily doses [3]. These associations between genotype and COAs response led the U.S. Food and Drug Administration (FDA) to release a warning in the warfarin insert to indicate the range of expected therapeutic warfarin dosages based on *CYP2C9* and *VKORC1* genotypes [8]. Furthermore, the clinical feasibility of incorporating *CYP2C9* and *VKORC1* genotyping-based COAs dosing regimens into routine clinical practice is being tested in large prospective clinical trials [9–11].

Substantial inter- and intraethnic variability in the frequencies of the *CYP2C9* and *VKORC1* alleles has been reported [5, 12]. The CYP2C9 *2 allele is absent in East Asian populations, whereas its frequency in African-Americans and Ethiopians has been estimated to be as low as 3.2% [5]. By contrast, a higher frequency of the CYP2C9 *2 allele (5%–19%) has been reported in Caucasians [5, 13]. Furthermore, the frequency of the CYP2C9 *3 allele is significantly lower in Asian populations (as low as 3.3%, compared to 4%–16% in Caucasians) [5, 13]. In Arabian populations, intraethnic variability in the frequency of the CYP2C9 *2 and *3 alleles has been reported, ranging from 7% to 21% and 3% to 9%, respectively [14–18]. In the case of the *VKORC1* –1639G>A polymorphism, interethnic variability in –1639AA frequency has also been reported [19]. Moreover, the –1639AA genotype, which is highly correlated with COAs sensitivity, is more common in Asian (frequency 80%) than Caucasian or African populations (estimated frequency 16%–25%) [19, 20]. Whereas the frequency of the –1639A COAs sensitivity allele ranges between 52% and 56% in the Arab populations studied to date [18, 21], no investigation of the *VKORC1* –1639G>A polymorphism in Saudi Arabians has been reported.

Few data are available concerning the prevalence of the CYP2C9 *2 and *3 alleles and the *VKORC1* –1639G>A polymorphism in distant populations of Saudi Arabia where social and in some areas religious beliefs favor consanguineous marriages and therefore limit genetic flow. In the Al-Ahsa region, which is part of the eastern province of Saudi Arabia, the rate of consanguineous marriage has been reported to be 59.1%, with marriages between first-degree relatives at 40% [22]. The reported inter- and intraethnic variability in the frequencies of the CYP2C9 *2 and *3 and *VKORC1* –1639A alleles was our rationale for studying the incidence of these variant alleles in the Al-Ahsa population. The results of this investigation will be critical for the coming era, in which genotype-guided dosing algorithms will be increasingly utilized to guide the prescription of COAs [9–11].

The aim of the present study was to investigate the frequency of the CYP2C9 *2 and *3 alleles and the *VKORC1* –1639G>A polymorphism as well as the number and percentages of individuals with genotypes predictive of COAs response in a representative sample of the population of Al-Ahsa, Saudi Arabia. We also sought to compare the data obtained with existing published data for other populations residing in a wider area of the Middle East. 

## 2. Materials and Methods

### 2.1. Subjects

All participants were of Arabian origin and residents of the Al-Ahsa urban area, which is located on the east coast of Saudi Arabia. The study protocol was approved by the Ethics Committee of King Faisal University, Hofouf, Saudi Arabia. All of the study participants were nonrelative volunteers and provided informed consent. A total of 131 healthy adult subjects (70 males and 61 females) were genotyped to determine the frequencies of the CYP2C9 *2 and *3 alleles and the *VKORC1* –1639G>A polymorphism. The mean (±SD) age of the subjects was 25 (±7) years (range: 19–52 years). The majority of the subjects (57%) reported to originate from a consanguineous marriage.

### 2.2. Genotyping

Genomic DNA was extracted from peripheral blood leukocytes using the QIAamp DNA Blood Mini Kit (Qiagen, Germany) according to the manufacturer's instructions. All subjects were genotyped for the CYP2C9 *2 and *3 alleles and the *VKORC1* –1639G>A polymorphism using PCR-restriction fragment length polymorphism (RFLP) protocols, as previously described [13, 20]. PCR amplifications were performed in duplicate by two independent researchers in an MJ Research PTC-200 thermocycler (Watertown, MA, USA). To ensure the accuracy of the results, an internal positive control was utilized for each polymorphism (rare allele) in each PCR-RFLP run.

### 2.3. Statistics

Data were analyzed using the Statistical Package for Social Sciences (SPSS) version 17.0 and are presented as the medians with 95% confidence intervals. Departure from the Hardy-Weinberg equilibrium was estimated using an exact 2-sided probability test using the formula provided by Weir [23]. Allele frequencies were compared to other ethnic population utilizing the two-tailed Fisher's exact test [24].

## 3. Results

The distributions of genotypes and alleles of CYP2C9 *2 and *3 and *VKORC1* –1639G>A polymorphisms in the studied population are shown in Table 1. 

For the CYP2C9 *2 and *3 alleles, 90 subjects (68.7%) were genotyped as CYP2C9 *1/*1, 35 subjects (26.7%) as CYP2C9 *1/*2, and 6 subjects (4.6%) as CYP2C9 *1/*3. The frequency of the CYP2C9 *2 and *3 alleles was estimated at 13.3% and 2.3%, respectively. None of the subjects were found to be homozygous or combined heterozygous for the CYP2C9 *2 and *3 alleles (CYP2C9 *2/*2, CYP2C9 *2/*3, and CYP2C9 *3/*3 genotypes). The genotype-derived PM phenotype was absent in the study population, whereas 68.7% of the population was predicted to be EM and 31.3% to be IM. 

For the *VKORC1* –1639G>A polymorphism, 49 subjects (37.4%) were genotyped as GG, 52 subjects (39.7%) as GA, and 30 subjects (22.9%) as AA. The frequency of the A allele was 42.7% (112 alleles). Consistent results for each genotype call were obtained by two researchers who performed the genotyping independently. 

Table 2 lists all of the combinations of the variant genotypes identified in the present cohort study. Of 49 subjects with the *VKORC1* –1639GG genotype, 35 subjects were genotyped as CYP2C9 *1/*1, 11 subjects as CYP2C9 *1/*2, and 3 subjects as CYP2C9 *1/*3. Among the 52 subjects with the *VKORC1* –1639GA genotype, 30 subjects were CYP2C9 *1/*1, 19 subjects were CYP2C9 *1/*2, and 3 subjects were CYP2C9 *1/*3. Among the 30 subjects with the *VKORC1* –1639AA genotype, 25 subjects were CYP2C9 *1/*1, 5 subjects were CYP2C9 *1/*2, and none carried the CYP2C9 *1/*3 genotype. 

According to the range of *CYP2C9-* and *VKORC1*-based warfarin dosages suggested by the U.S. FDA [8], 58% of our population (those carrying the genotypes *VKORC1* –1639GG or 1639GA and CYP2C9 *1/*1, or *VKORC1* –1639GG and CYP2C9 *1/*2) would require higher dosages (5–7 mg). In contrast, the remainder of the studied subjects (42%; those carrying the genotypes *VKORC1* –1639GG and CYP2C9 *1/*3, *VKORC1* –1639GA and CYP2C9 *1/*2 or CYP2C9 *1/*3, or *VKORC1* –1639AA and CYP2C9 *1/*1 or CYP2C9 *1/*2) may require intermediate dosages (3-4 mg). Due to the lack of CYP2C9 *2/*2 or CYP2C9 *3/*3 genotypes and the lack of the combined genotype *VKORC1* –1639AA and CYP2C9 *1/*3, none of the studied subjects belonged to the sensitive group that would require low warfarin dosages (range of 0.5–2 mg).

We also analyzed the data for potential gender differences. However, there were no significant differences in the genotype frequencies of the CYP2C9 *2 and *3 alleles and the *VKORC1* –1639G>A polymorphism in our study group of 131 Saudi Arabian subjects (70 male and 61 female) (data not shown). 

Finally, to investigate possible differences between individuals who originated from consanguineous marriages and those who did not, we analyzed the distribution of the CYP2C9 *2 and *3 alleles and the *VKORC1* –1639G>A polymorphism according to this factor. However, the frequencies of the genotypes and alleles studied did not differ with respect to origin from a consanguineous marriage (data not shown).

## 4. Discussion

The pharmacogenomics of COAs is one field that is most ready to apply genotype-guided dosing in clinical practice. It has been estimated that polymorphisms in the *CYP2C9* and *VKORC1* genes together account for 35%–50% of the variability in COAs initiation and maintenance dosage requirements [20, 40]. Thus, efforts are focused on incorporating this knowledge into the dosing regimens currently used, and genotype-based algorithms are currently being tested in large randomized trials to validate the accuracy, safety, and cost effectiveness of incorporating *CYP2C9* and *VKORC1* genotypes into the optimization of anticoagulant therapy [3, 9]. 

In the era of developing and testing genotype-guided COAs dosing algorithms, there remain populations in which the frequency of the major *CYP2C9* and *VKORC1* polymorphisms has not been assessed. Thus, potential differences in the prevalence of *CYP2C9* and *VKORC1* genetic variants in different populations may lead to adjustments of genotype-based COAs dosing algorithms or may serve as motivation to identify novel genetic variants that influence COAs therapeutic responses. Towards this goal, the current study reported the frequency of the CYP2C9 *2 and *3 alleles and *VKORC1* –1639G>A polymorphism in a distant population residing on the east coast of Saudi Arabia. Some of the *CYP2C9* variants that lead to decreased enzymatic activity, such as CYP2C9 *5, *6, and *11, were not included in this study because they have not been observed in Caucasian or Middle Eastern populations, in contrast to their higher frequency in African populations. Although consanguineous marriage is traditionally favored among Saudis, genetic inflow in Al-Ahsa is further limited due to social and religious beliefs, which could have led to the differences in the prevalence of the *CYP2C9* and* VKORC1* genotypes and alleles studied.

In general, the genetic characteristics of Arabian populations are not well characterized. In the case of *CYP2C9*, there are scattered reports on *CYP2C9* frequencies in Arabs in general and Saudis in particular, whereas to the best of our knowledge, this is the first report of the frequency of the *VKORC1* –1639G>A polymorphism in a population in Saudi Arabia. Among Arabs in general, there exists great intraethnic variability in the frequencies of the CYP2C9 *2 and *3 alleles. In Saudi Arabia, Mirghani et al. [14] reported that the frequencies of the CYP2C9 *2 and *3 alleles among Saudis residing in Riyadh were similar to those in Caucasian populations (11.7% and 9.1%, resp.) [14]. Unpredictably, the frequency of CYP2C9 *3 was significantly different from the frequency of *3 in our study group (Table 3). In the Omani population, CYP2C9 *2 and *3 allele frequencies are markedly lower and have been estimated at 7.4% and 2.9%, respectively [17]. The later was the closest Among Arab populations to the frequency of CYP2C9 *3 in our study subjects (2.3%). In the Egyptian population, the frequencies of the CYP2C9 *2 and *3 alleles have been estimated to be 12% and 6%, respectively [16]. In Lebanese individuals, the frequencies of CYP2C9 *2 and *3 have been reported to be 11.2% and 9.6%, respectively [18]. 

 In our study population, the CYP2C9 *2 allele frequency (13.4%) was similar to that reported for other Arabian and Caucasian populations (Table 3). However, we found a significantly reduced frequency of the CYP2C9 *3 allele (2.3%). In addition, genotypes predicting the CYP2C9 PM phenotype (i.e., CYP2C9 *2/*2, CYP2C9 *2/*3, and CYP2C9 *3/*3) were absent in the subjects studied. One possible explanation for this finding is that our study population comprised residents of Al-Ahsa, where the population can be divided based on religious beliefs into two main populations (Sunni and Shiaah) between which intermarriage rarely occurs. Moreover, within each of the two populations, social customs may further limit intermarriage between nonrelatives, further resulting in decreased genetic inflow.

Although we did not find statistically significant differences in the distribution of CYP2C9 *2 and *3 alleles and *VKORC1* –1639G>A polymorphisms in our study group according to presence or absence of consanguineous marriage, different frequencies in variants predicting low *CYP2C9* enzymatic activity should be expected in similar populations elsewhere in Saudi Arabia.

 Regarding the *VKORC1* –1639G>A polymorphism, we found that the frequencies of genotypes and alleles were similar to those reported in Caucasian populations. The *VKORC1* –1639AA genotype and *VKORC1* –1639A allele frequencies were estimated to be 22.9% and 42.7%, respectively, which suggests that approximately 23% of the studied population is sensitive to COAs and would require lower dosages of COAs. However, this finding should not be generalized to all Saudi Arabians prior to assessing the relative genotype frequencies in populations residing in different regions with different social backgrounds. Indeed, we found that the frequency of the *VKORC1* –1639A allele was lower than that reported for other Arabian populations (i.e., 52.4% in the Lebanese population) [18]. 

The relatively low frequencies of the CYP2C9 *3 allele and *CYP2C9* genotype-derived PMs indicate that, in this population, COAs dosage adjustments and responses may depend more on *VKORC1* gene polymorphisms. This finding is of utmost importance for personalizing COAs therapy in the Al-Ahsa region. The presence of other rare alleles that cause reduced CYP2C9 activity and could potentially interfere with COAs response requires further investigation. In addition to COAs metabolism, normal to slightly decreased metabolism of other drugs that are CYP2C9 substrates would be expected in the studied population in addition to the incidence of adverse effects due to diminished metabolism, as is the case with antidiabetic drugs metabolized by CYP2C9 [41]. 

In conclusion, we report that the frequency of the CYP2C9 *3 allele varies substantially among Saudi Arabian populations. In light of the frequency of these genetic variants, the *VKORC1* –1639G>A polymorphism may be the major determinant of COAs pharmacogenomics in the studied population. Overall, it appears that some *CYP2C9* genotypes known to be associated with sensitivity to COAs are less common in the studied population, particularly CYP2C9 *3. Investigation of the frequencies of other *CYP2C9* alleles in this population as well as other Saudi populations, especially those variants known to be associated with decreased levels of the CYP2C9 enzyme, such as CYP2C9 *5, *8, *11, *13–18, *30, and *33, is recommended. To apply COA pharmacogenomics in clinical practice in Saudi Arabia, we need to understand the frequencies of genetic variants in various Saudi Arabian populations in order to facilitate clinical decision making and improve patient management. 

## Figures and Tables

**Table 1:** Frequencies of the *CYP2C*9*2 and *3 and *VKORC1* –1639G>A genotypes and alleles in a sample of the Saudi Arabian population (n = 131).

Genotypes and alleles	No. of individuals, relative frequency, and 95% confidence intervals	
	n (%)	95% CI
*CYP2C*9 genotype		
*CYP2C*9*1/*1	90 (68.7)	60.4–76.2
CYP2C9 *1/*2	35 (26.7)	19.7–34.7
*CYP2C*9*1/*3	6 (4.6)	1.9–9.2
*CYP2C*9*2/*2	0	—
*CYP2C*9*2/*3	0	—
*CYP2C*9*3/*3	0	—
*CYP2C*9 allele		
*CYP2C*9*2	35 (13.3)	9.7–17.9
*CYP2C*9*3	6 (2.3)	1.0–4.7
*VKORC1* genotype		
GG	49 (37.4)	29.5–45.9
GA	52 (39.7)	31.6–48.2
AA	30 (22.9)	16.4–30.6
*VKORC1* allele		
G	150 (57.3)	51.2–63.1
A	112 (42.7)	36.9–48.8

**Table 2:** Combination of *CYP2C*9 and *VKORC1* genotypes in a sample of the Saudi Arabian population (n = 131).

*CYP2C*9 genotype	*VKORC1* genotype			Total
	GG, *n* (%)	GA, *n* (%)	AA, *n* (%)
*CYP2C*9*1/*1	35 (26.7)	30 (22.9)	25 (19.1)	90 (68.7)
*CYP2C*9*1/*2	11 (8.4)	19 (14.5)	5 (3.8)	35 (26.7)
*CYP2C*9*1/*3	3 (2.3)	3 (2.3)	—	6 (4.6)
Total	49 (37.4)	52 (39.7)	30 (22.9)	131 (100)

**Table 3:** Prevalence of CYP2C9 *2 and *3 in different ethnic groups.

Population	Allele frequencies of *CYP2C9 *					Ref.
	*N*	*2	(*P*)	*3	(*P*)
Middle East Arab						
Saudi (Al-Ahsa)	131	0.133		0.023		Current
Saudi (Riyadh)	192	0.117	0.73	0.091	0.03	[14]
Egyptian	247	0.120	0.87	0.060	0.13	[16]
Jordanian	263	0.135	1.0	0.068	0.09	[25]
Lebanese	161	0.112	0.72	0.096	0.03	[18]
Omani	189	0.074	0.18	0.029	0.72	[17]
Caucasian						
American	100	0.080	0.29	0.060	0.19	[26]
Croatian	200	0.165	0.53	0.095	0.02	[27]
German	118	0.140	0.86	0.050	0.32	[28]
Greek	283	0.129	1.0	0.081	0.03	[13]
Italian	157	0.112	0.86	0.092	0.03	[29]
Turkish	499	0.106	0.54	0.100	0.006	[30]
Belgian	121	0.10	0.56	0.074	0.081	[31]
Asian						
Japanese	218	0	<0.0001	0.021	0.72	[32]
Korean	574	0	<0.0001	0.011	0.22	[33]
Chinese (Mongolian)	280	0	<0.0001	0.03	1.0	[34]
Vietnamese	157	0	<0.0001	0.022	1.0	[35]
Malaysian (Malay)	202	0.019	0.0003	0.024	1.0	[36]
African						
Beninese	111	0	<0.0001	0	<0.0001	[31]
Ethiopian	150	0.04	0.02	0.02	1.0	[29]
Ghanaian	204	0	<0.0001	0	0.06	[37]
Iranian	200	0.13	1.0	0	0.06	[38]
African-American	490	0.011	<0.0001	0.018	0.48	[39]
